# Natural Products Inhibit Bacterial Growth and Modulate Quorum‐Sensing Systems *Las* and *Rhl* in *Pseudomonas aeruginosa* PA14

**DOI:** 10.1002/cbdv.202502040

**Published:** 2025-10-03

**Authors:** Oswaldo Pablo Martínez‐Rodríguez, Rodolfo García‐Contreras, Rodrigo Aguayo‐Ortiz, Itzel Rubí Yeverino, Carlos A. Fajardo‐Hernández, Albert D. Patiño, Hugo A. Hernández Pérez, Mariel Hernández‐Garnica, Mario Figueroa

**Affiliations:** ^1^ Departamento De Farmacia, Facultad de Química Universidad Nacional Autónoma De México Ciudad de México México; ^2^ Departamento De Microbiología y Parasitología Facultad De Medicina, Universidad Nacional Autónoma de México Ciudad de México México; ^3^ Departamento De Biología, Facultad de Química Universidad Nacional Autónoma De México Ciudad de México México

**Keywords:** LasR and RhlR proteins, natural products library, *Pseudomonas aeruginosa*, quorum sensing

## Abstract

Quorum sensing (QS) is a bacterial cell communication system in *Pseudomonas aeruginosa*, which regulates the production of several virulence factors, including pyocyanin, exoproteases, and biofilm formation. Natural products (NPs) have demonstrated their ability to control QS in different bacteria. This work evaluated the antimicrobial and anti‐QS potential of a small library of NPs from Mexican biodiversity against *P. aeruginosa* PA14. Alternariol 4‐methyl ether (**12**), iridoskyrin (**17**), and 5,8‐epi‐dioxyergosta‐6,9(11),22‐trien‐3‐ol (**39**) showed the most potent antimicrobial activity at both test concentrations. Regarding anti‐QS activity, fuscin (**13**), neosartorin (**18**), 8ʹ‐hydroxy zearalenone (**21**), penicillic acid (**26**), 5,6‐dehydroxypenicillic acid (**27**), pitholide B (**36**), and citrinin (**38**) inhibited pyocyanin production, exoproteases activity, and biofilm formation at the lowest concentration without significantly altering growth. Subsequently, in silico studies of deep‐learning molecular docking and molecular dynamics indicated the putative mechanism of action of these compounds through their binding to the ligand‐binding domains of LasR and RhlR, target proteins that control QS systems. These results demonstrate that NPs, such as pitholide B (**36**) and citrinin (**38**), are potential candidates against *P. aeruginosa*.

## Graphical abstract

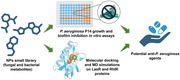

## 1 Introduction

The World Health Organization (WHO) classifies *Pseudomonas aeruginosa* as a high‐priority pathogen [1]. This bacterium readily infects wounds and burns, causes acute pulmonary and urinary tract infections, and leads to cystic fibrosis in immunocompromised individuals, resulting in devastating clinical infections worldwide. The natural resistance of *P. aeruginosa* triggers the rapid development of multidrug resistance (MDR) due to the overexpression of genes encoding proteins responsible for resistance, leading to the emergence of numerous MDR strains [2, 3, 4, 5].

One key mechanism underlying the resistance of P. aeruginosa and other bacteria is quorum sensing (QS), a communication process that depends on cell density. In this process, signaling molecules, called autoinducers, are released into the microenvironment to detect the presence of other cells and coordinate the activities of the bacterial population. This allows the expression of genes that control the production of various virulence factors; therefore, QS inhibition is crucial in addressing MDR [6, 7].

*P. aeruginosa* has two QS systems mediated by *N*‐acyl homoserine lactones (AHLs), autoinducers *Las* and *Rhl*. The *Las* system consists of LasI synthase, LasR receptor, and the *N*‐3‐oxo‐dodecanoyl‐L homoserine lactone autoinducer. The *Rhl* system consists of RhlI synthase, RhlR receptor, and the *N*‐butyryl homoserine lactone autoinducer [8]. During QS, *P. aeruginosa* releases AHLs into its environment, which are recognized by the receptors of other bacteria. This regulates both the production of virulence factors and biofilm formation [9], allowing the accumulation of high amounts of β‐lactamases that hydrolyze antibiotics before they reach their target [10]. The *Las* system regulates the *Rhl* system and virulence factors, including exoproteases, as well as the type II secretion system. The *Rhl* system enhances the production of rhamnolipids, hydrogen cyanide, and pyocyanin [8, 11]. Specifically, pyocyanin is a redox‐active blue pigment responsible for producing reactive oxygen species, such as hydrogen peroxide, capable of depleting antioxidant reserves such as glutathione, thus causing oxidative damage to the host [6, 11]. In addition, *P. aeruginosa* produces several proteases, including elastase B, alkaline protease A, protease IV, and *Pseudomonas* small protease, which are capable of inactivating or degrading components of the complement system. Exoproteases play a key role in the initial colonization of tissues, as their production increases during acute infections [12].

Given the significant role of QS in *Pseudomonas* pathogenicity, targeting this communication process is a promising strategy to mitigate the severity of infections. The use of anti‐QS and antibiofilm compounds is a potential alternative to traditional antibiotics. Fungi and bacteria are excellent sources of specialized natural products (NPs) with unique structural diversity and potent antimicrobial activity, making them ideal for discovering and developing new drugs [13, 14, 15]. These metabolites have evolved due to various anthropogenic factors to which the producing organisms are exposed, resulting in privileged structures with unique chemical characteristics that favor ligand‐protein interactions in target organisms. Natural metabolites thus provide a valuable source of bioactive compounds that can interfere with QS pathways and biofilm formation.

The literature provides numerous examples of screening libraries of crude extracts and fractions to discover novel antimicrobial NPs [16, 17]. However, testing of libraries of pure NPs is not often reported, and repositioning these molecules could lead to the discovery of potential new drugs. Thus, as part of our continuous effort to discover new chemical diversity with antimicrobial potential from unexplored Mexican biodiversity, we recently evaluated the antibacterial and antibiofilm activity of a small molecule library of NPs against methicillin‐sensitive *Staphylococcus aureus* (MSSA) and methicillin‐resistant *S. aureus* (MRSA), and determined their putative molecular interaction with AgrA and SarA, proteins involved in biofilm formation [18]. From this epifiscalin C, fiscalin C, dimethylgliotoxin, aspernolide B, and butyrolactones I and IV showed important antibiofilm potential with likely molecular interactions with AgrA and SarA proteins.

In this study, we focused on the impact of the small library of NPs on the growth, virulence factors, and biofilm formation of *P. aeruginosa*. Additionally, in silico studies were conducted to investigate the potential mechanisms by which these compounds may interfere with QS pathways, providing new insights into the development of alternative therapeutic strategies for combating *P. aeruginosa* infections.

## 2 Experimental

### 2.1 Tested Compounds

The small library of NPs used in this study (Table S1) were isolated from fungal and bacterial extracts with antibacterial activity (≥80% growth inhibition) at both 20 and 200 µg/mL against at least one of the following ESKAPE bacteria (data not shown): [19] *Enterococcus faecalis* (ATCC 29212), MSSA and MRSA (MSSA ATCC 25923 and MRSA ATCC 43300), *Klebsiella aerogenes* (ATCC 13048), *Klebsiella quasipneumoniae* (ATCC 700603), *Acinetobacter baumannii* (ATCC 17978 and 564 multidrug resistant clinical isolate), *P. aeruginosa* (ATCC 27853), and *Enterobacter hormaechei* (ATCC 700323). Stock solutions of each tested compound (1 mg/mL in dimethyl sulfoxide [DMSO]) were prepared and maintained at 4°C until use.

### 2.2 Growth Inhibition

*P. aeruginosa* PA14 strain was seeded on Luria‐Bertani (LB) agar and incubated at 37°C for 24 h. Next, overnight precultures of 1 CFU were prepared in 5 mL of LB and incubated aerobically at 200 rpm for 24 h at 37°C. After this time, overnight growth was determined by measurement of optical density (OD) at 600 nm in a microplate reader (VICTOR Nivo, PerkinElmer, Waltham, MA). The strain was then inoculated into a final volume of 1 mL LB at 0.05 OD*_i_* with and without the NPs at 16 and 64 µg/mL at 200 rpm and 37°C for 20 h. Vehicle (DMSO 1.6%) was used as a negative control, and quercetin 50 µM was used as a QS positive control [20]. After incubation, the growth (OD_600nm_) was determined in a microplate reader.

In the case of the three compounds with the strongest inhibitory effects on QS‐dependent virulence factors, 5,6‐dehydroxypenicillic acid (**27**), pitholide B (**36**), and citrinin (**38**), *P. aeruginosa* PA14 growth curves were done with three independent cultures, recording growth (OD_600nm_) every 30 min for 20 h in a microplate reader (VICTOR Nivo). Results are expressed in averages ± standard deviation.

### 2.3 Determination of Virulence Factors: Pyocyanin, Exoprotease Activity, and Biofilm Inhibition

Pyocyanin production was measured according to López‐Jácome et al. [8]. After determining the growth inhibition of the cultures treated with or without the compounds of interest, the cultures were centrifuged at 13 000 rpm for 4 min. Then, 400 µL of supernatant was added to 210 µL of CHCl_3_ and vortexed for 3 min. The mixture was centrifuged for 4 min at 13 000 rpm. 200 µL of the organic phase was recovered and mixed with 400 µL of 0.2 N HCl by vortexing and then centrifuged at 13 000 rpm for 4 min. The aqueous phase (pyocyanin) was recovered, and the absorbance was measured at 520 nm in a microplate reader.

Exoprotease activity was measured by enzymatic degradation of azocasein (proteolytic substrate), as reported by Khatun et al. [21]. Briefly, 10 µL of the previously recovered supernatant was inoculated with 90 µL of azocasein and incubated at 37°C for 40 min. After incubation, 50 µL of the reaction was mixed with 200 µL of 1% acetic acid by vortexing and then centrifuged at 13 000 rpm for 4 min. A 50 µL aliquot was taken from the supernatant, and the reaction was stopped with 0.5 N NaOH. The absorbance was subsequently read at 405 nm in a microplate reader.

Inhibition of *P. aeruginosa* PA14 biofilms was assessed in a 48‐well microplate according to García‐Lara et al. [22]. Briefly, each well was inoculated with overnight cultures at 0.2 OD*_i_* in LB with or without the compounds of interest (16 and 64 µg/mL) in triplicate to a final volume of 500 µL and incubated aerobically at 37°C for 24 h. The negative control was the vehicle (DMSO 1.6%), and the positive control was quercetin 50 µM [20]. After incubation, planktonic bacterial growth was measured by reading the OD_600nm_. The supernatant was removed, and the wells were washed with 500 µL distilled water and dried. The biofilms were fixed in absolute methanol for 20 min in the dark, and then the methanol was discarded. The biofilms were stained with 1% crystal violet and incubated at 37°C for 40 min. The wells were washed twice with distilled water, and then the dye was extracted with 500 µL of absolute ethanol and incubated for 20 min. The absorbance was determined at 570 nm in a microplate reader.

Bacterial growth, virulence factors, and biofilm inhibition percentages were obtained using the following formula:

$$\mathrm{Inhibition}\;(\%)=\left(\frac{\mathrm{Control}\;\mathrm{negative}\;OD_{\mathrm{nm}}-\mathrm{Test}\;OD_{\mathrm{nm}}}{\mathrm{Control}\;\mathrm{negative}\;OD_{\mathrm{nm}}}\right)\;\times \;100$$

### 2.4 Statistical Analysis

The results of growth inhibition, virulence factors, and biofilm inhibition were analyzed in GraphPad Prism v.9.4.1 (Boston, MA), using Dunnett's multiple means test with a *p* ≤ 0.5.

### 2.5 In Silico Analysis

#### 2.5.1 Ligand and Protein Preparation

Ligand 3D structures were optimized through two energy minimization steps: first with the Universal Force Field in OpenBabel [23], then with Gaussian 16 [24] using B3LYP/6‐31G(d) [25, 26]. Protein crystal structures of PaLasI (PDB: 1ro5) [27], PaLasR (PDB: 2uv0) [28], and PaRhlR (PDB: 8dq0) [29] were obtained from the PDB and prepared using UCSF Chimera v1.17.1 [30].

#### 2.5.2 Molecular Docking and Molecular Dynamics Simulations

Docking was performed with DiffDock [31], generating 40 poses per protein‐ligand complex (20 inference steps, batch size 5, 18 total steps). The highest‐confidence poses were selected for molecular dynamics (MD) simulations. Then, ligands were parameterized with ACPYPE (AMBER framework) [32, 33, 34], and MD simulations were run in GROMACS 2021 [35] using the AMBER14sb [36] force field. Each protein‐ligand complex was solvated (TIP3P water, 0.15 M NaCl), energy‐minimized, equilibrated (1 ns NVT + 1 ns NPT), and subjected to a 100 ns NPT production run (300 K, 1 bar). Constraints and cutoffs included: LINCS for H‐bonds [37, 38, 39], LJ potential (1.0–1.2 nm), PME for long‐range electrostatics [40].

#### 2.5.3 Trajectory Analysis

From the last 50 ns, RMSD, RMSF, protein‐ligand contacts (Q), and binding free energy (ΔG_bind, via MM/GBSA with gmx_mmpbsa) [41] were calculated. Clustering based on ligand RMSD was used to identify the most representative binding pose (Figures S2–S4).

## 3 Results and Discussion

Among the NPs evaluated against the growth of *P. aeruginosa* PA14 (Table S2), alternariol 4‐methyl ether (**12**) and iridoskyrin (**17**) achieved an inhibitory effect (≥40%) at 16 µg/mL and 64 µg/mL (Table 1); epi‐pestalamide A (**3**), meleagrin A (**5**), atranone B (**6**), fuscin (**13**), monorden A (**22**), cholic acid (**23**), 8‐methoxynaphtalen (**30**), 1,8‐dimethoxynaphtalen (**30**), 5‐carboxy‐methylmellein (**32**), 9‐methoxy‐6,9a‐dimethyl‐3‐octanoyl‐2‐oxo‐9,9a‐dihydro‐2*H*‐furo[3,2‐g]benzopyran‐4‐carbaldehyde (**35**), pitholide D (**37**), Sch‐378161 (**40**), and beauvericin (**42**) exhibited a concentration‐dependent bacterial growth inhibitory effect between 20% and 60%; and 5,8‐epi‐dioxyergosta‐6,9(11),22‐trien‐3‐ol (**39**) was the only compound with antimicrobial activity of over 70% at both test concentrations (Table 1).

**TABLE 1:** Compounds with antimicrobial activity on *P. aeruginosa* PA14 at 16 and 64 µg/mL.

Compound	Growth inhibition (%)	
	16 µg/mL	64 µg/mL
LB (negative control)	0.0±3.3	
DMSO 1.6% (vehicle)	1.2±5.2	
Epi‐pestalamide A (**3**)	34.2±2.2	40.1±4.0
Meleagrin A (**5**)	30.1±1.9	43.9±5.1
Atranone B (**6**)	34.9±2.7	57.1±1.8
Alternariol 4‐methyl ether (**12**)	52.4±0.8	65.0±0.8
Fuscin (**13**)	8.4±2.7	53.1±8.2
Iridoskyrin (**17**)	44.0±2.9	54.4±3.1
Monorden A (**22**)	20.7±4.5	40.7±3.3
Cholic acid (**23**)	25.0±2.4	54.4±2.0
1,8‐Dimethoxynaphthalen (**30**)	21.7±2.8	48.1±2.0
9‐Methoxy‐6,9*a*‐dimethyl‐3‐octanoyl‐2‐oxo‐9,9*a*‐dihydro‐2*H*‐furo[3,2‐*g*]benzopiran‐4‐carbaldehyde (**35**)	36.9±5.2	49.5±5.2
Pitholide D (**37**)	30.4±3.7	47.1±2.6
5,8‐Epi‐dioxyergosta‐6,9(11),22‐trien‐3‐ol (**39**)	70.5±9.7	83.5±5.2
Sch‐378161 (**40**)	29.7±1.5	35.6±2.6
Beauvericin (**42**)	28.4±4.3	40.2±4.5

**FIGURE 1:**
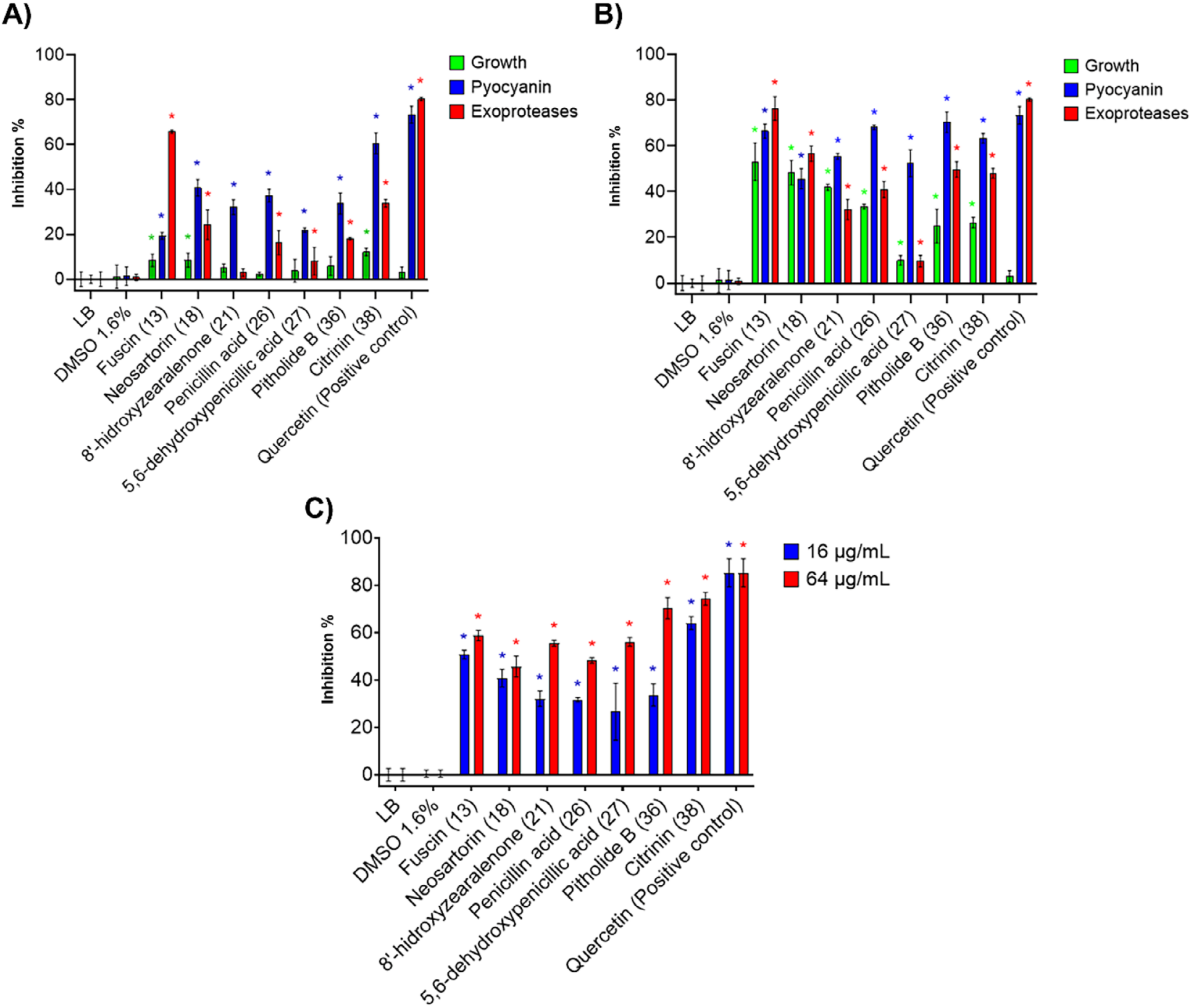
Inhibition of pyocyanin production and exoproteases’ activity in *P. aeruginosa* PA14 using fungal metabolites at (A) 16 µg/mL and (B) 64 µg/mL at 20 h. (C) Inhibition of biofilm formation at 24 h. Luria‐Bertani (LB) broth: negative control; dimethyl sulfoxide (DMSO): vehicle (1.6%); quercetin 50 µg/mL: positive control QS. *Statistically significant differences with respect to the negative control; *p* ≤ 0.05 according to Dunnett's mean test, *n* = 3.

After determining the antimicrobial activity of the NP library, we evaluated the effect of the NPs that showed bacterial growth inhibitory <20% on the expression of QS‐controlled virulence factors of *P. aeruginosa* PA14, including pyocyanin production, exoprotease activity, and biofilm formation (Figure 1). It is worth noting that compounds that did not show relevant antimicrobial activity but inhibit these virulence factors may be considered QS modulators.

The inhibition of pyocyanin production was over 30% at 16 µg/mL for fuscin (**13**), neosartorin (**18**), 8ʹ‐hydroxyzearalenone (**21**), penicillic acid (**26**) [42], 5,6‐dehydroxypenicillic acid (**27**), and pitholide B (**36**) (Figure 1A). The highest inhibitory activity was that of citrinin (**38**) (60.6%). At 64 µg/mL (*Figure* *1B*), these compounds also inhibited the pyocyanin production, but growth inhibition was observed. Thus, the inhibition of this virulence factor was due to a reduction in growth. Only 5,6‐dehydroxypenicillic acid (**27**), pitholide B (**36**), and citrinin (**38**) showed significant inhibition of this virulence factor (≥60%) without significant effects on growth (Figure S1).

The inhibition of the proteolytic activity of exoproteases is also crucial to control the virulence and pathogenicity of *P. aeruginosa*. Thus, casein was used as a proteolytic substrate to evaluate the effect of the NP library against this virulence factor. At 16 µg/mL, fuscin (**13**) showed ≥60% inhibition without affecting bacterial growth; it was followed by citrinin (**38**) with 35% inhibition (Figure 1A). Epi‐pestalamide A (**3**), aspochacalin D (**4**), myrotecisin B (**9**), equisetin (**10**), and iridoskyrin (**18**) inhibited casein proteolysis (≥50%), but growth was also significantly inhibited. At 64 µg/mL, pitholide B (**36**) and citrinin (**38**) inhibited exoproteases activity by 49.7% and 48.4%, respectively, without significantly affecting growth (Figure 1B). Some of the compounds from the NP library showed slight or no inhibition of the proteolytic activity of exoproteases (≤10%): meleagrin A (**5**), atranone B **(6**), viridicatin (**7**), alternariol 4‐methyl ether (**12**), zearalenone (**20**), monorden A (**22**), cholic acid (**23**), helvolic acid (**24**), diorcinol (**28**) and 1,8‐dimethoxynaphthalen (**30**); however, as mentioned above, these compounds had antimicrobial activity (Table 1).

The inhibition of *P. aeruginosa* biofilm formation was also evaluated. Although equisetin (**10**), fusarubin (**14**), przwalsquinone B (**15**), hirsutelic acid (**33**), Sch‐217040 (**41**), and beauvericin (**42**) strongly inhibited the biofilm formation (≥50%) of strain PA14 at both test concentrations, antimicrobial activity was also observed. The compounds that significantly inhibited both pyocyanin production and exoprotease activity also demonstrated the ability to inhibit biofilm formation in a concentration‐dependent manner (Figure 1C).

**FIGURE 2:**
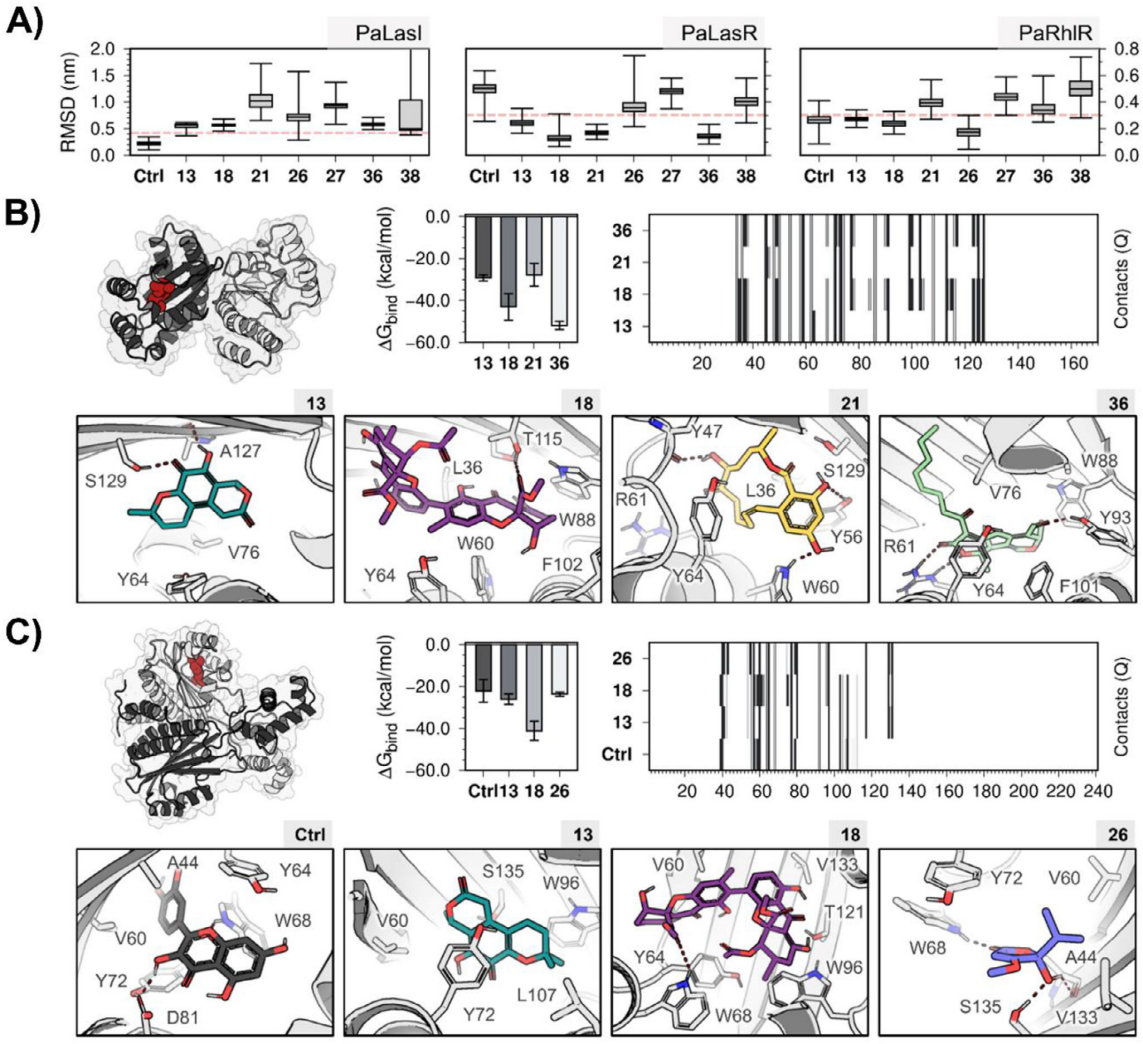
Analysis of the last 50 ns of the protein‐ligand complexes molecular dynamics (MD) simulations. (A) Distribution analysis of ligand root mean square deviation (RMSD) in PaLasI, PaLasR, and PaRhlR. Depiction of the ligand‐binding site (red), binding free energy (∆G_bind_), number of contacts (Q), and the most representative binding mode of ligands with RMSD values lower than 0.3 nm in (B) PaLasR and (C) PaRhlR.

These results demonstrate that fuscin (**13**), neosartorin (**18**), 8ʹ‐hydroxyzearalenone (**21**), penicillic acid (**26**) [36], 5,6‐dehydroxypenicillic acid (**27**), pitholide B (**36**), and citrinin (**38**) are capable of interfering with the QS systems of *P. aeruginosa* by inhibiting pyocyanin production, exoprotease activity, and biofilm formation. To elucidate the putative mechanism of action of these compounds, a series of in silico analyses was performed, including molecular docking and stability of ligand‐protein complexes by MD simulations (Figure 2 and Tables 3 and 4). The target proteins LasI, LasR, and RhlR, which regulate the production of virulence factors and biofilm formation [9], were selected at the top of the hierarchy of *P. aeruginosa* QS systems.

**TABLE 3:** Interactions fraction of hydrogen bonds and hydrophobic interactions of the best compounds with the amino acids comprising the binding site of LasR and RhlR.

Ligand	Interaction fraction (IF)	
	Hydrogen bonds	Hydrophobic interactions
LasR		
Fuscin (13)	S129 (0.90); A127 (0.85)	Y64 (0.84); V76 (0.57); I52 (0.35); W88 (0.32); L40 (0.28); L36 (0.28)
Neosartorin (18)	T115 (0.91)	W88 (0.92); T115 (0.89); Y64 (0.70); L36 (0.67); F102 (0.60); W60 (0.59); L125 (0.52); V76 (0.37); Y47 (0.30); R61 (0.28); A127 (0.26)
8ʹ‐Hydroxyzearalenone (21)	Y47 (0.79); W60 (0.67); Y56 (0.56)	Y64 (1.00); A127 (0.74); R61 (0.73); Y56 (0.71); L36 (0.70); W88 (0.67); T115 (0.53); Y47 (0.45); F101 (0.42); W60 (0.26)
Penicillin acid (26)	R61 (0.43)	Y64 (0.49); L36 (0.48); V76 (0.40); A50 (0.30); I52 (0.25); Y47 (0.25)
5,6‐Deshydroxypenicillic acid (27)	W60 (0.97)	A105 (0.99); F101 (0.96); W88 (0.88); Y64 (0.75); F102 (0.31); Y93 (0.25)
Pitholide B (36)	Y93 (0.91); R61 (0.88)	Y64 (1.00); W88 (0.84); F101 (0.81); L40 (0.39); V76 (0.36); W60 (0.33); I52 (0.32); L125 (0.32); T80 (0.28)
Citrinin (38)	R61 (1.00)	W88 (0.90); Y64 (0.74); F102 (0.67); F101 (0.56); L110 (0.47); Y56 (0.40); L36 (0.27)
Quercetin (positive control)	Y47 (0.68)	L36 (0.78); Y64 (0.77); I52 (0.70); V76 (0.67); L40 (0.45); Y47 (0.42)

RhlR		
Fuscin (13)	—	Y72 (0.92); W96 (0.65); L107 (0.53); F101 (0.30); A111 (0.27); A44 (0.26)
Neosartorin (18)	W68 (0.82)	Y64 (0.98); W96 (0.79); V60 (0.72); I84 (0.54); V133 (0.53); F101 (0.51); L107 (0.34); A111 (0.33); W68 (0.29); L69 (0.27)
8ʹ‐Hydroxyzearalenone (21)	D81 (0.99); W68 (0.91)	Y72 (0.99); Y64 (0.88); F101 (0.83); W96 (0.78); W68 (0.54); L116 (0.47); L69 (0.38); L107 (0.34)
Penicillin acid (26)	W68 (0.95); S135 (0.91); A44 (0.59)	Y72 (0.66); V133 (0.56); V60 (0.58); I84 (0.36); A44 (0.36)
5,6‐Deshydroxypenicillic acid (27)	S135 (1.00); T121 (0.56); Y72 (0.29)	V133 (0.78); V60 (0.47); Y72 (0.47); A44 (0.35); T121 (0.33); T58 (0.27)
Pitholide B (36)	W68 (0.60)	Y72 (0.89); V60 (0.63); I84 (0.57); A44 (0.55); P56 (0.42); T58 (0.41)
Citrinin (38)	—	F101 (0.92); W96 (0.88); Y72 (0.63); A111 (0.44); W108 (0.43); L116 (0.32)
Quercetin (positive control)	D81 (0.54); W68 (0.40); L107 (0.31)	Y64 (0.99); V60 (0.95); Y72 (0.90); A44 (0.87); W68 (0.79); W96 (0.52); L107 (0.31)

**TABLE 4:** Docking score values computed with Uni‐GBSA.

Compound	PaLasI		PaLasR		PaRhlR	
	Score (kcal/mol)	Ligand Efficiency	Score (kcal/mol)	Ligand Efficiency	Score (kcal/mol)	Ligand Efficiency
Fuscin (13)	−19.23	0.96	−28.95	1.45	−32.52	1.63
Neosartorin (18)	−26.32	0.54	−11.97	0.24	11.06	−0.23
8ʹ‐Hydroxyzearalenone (21)	−10.58	0.44	−41.74	1.74	−39.81	1.66
Penicillin acid (26)	−17.41	1.45	−18.26	1.52	−23.30	1.94
5,6‐Deshydroxypenicillic acid (27)	−9.11	0.76	−17.63	1.47	−18.27	1.52
Pitholide B (36)	−29.32	1.05	−21.08	0.75	−41.01	1.46
Citrinin (38)	−2.75	0.15	−30.90	1.72	−28.76	1.60
Quercetin (Positive control)	−4.64	0.21	−41.24	1.87	−33.31	1.51

Figure 2A depicts the ligand RMSD distribution during the last 50 ns of simulation. Except for quercetin (positive control), none of the compounds exhibited an RMSD lower than 0.3 nm in LasI. In contrast, at least four different compounds met this threshold in LasR and RhlR. Consequently, we decided to exclude LasI as a therapeutic target for this study, focusing the analysis on the complexes formed with LasR and RhlR.

In the homodimer of the ligand‐binding domain (LBD) of LasR, fuscin (**13**), neosartorin (**18**), 8ʹ‐hydroxyzearalenone (**21**), and pitholide B (**36**) showed binding with the autoinducer site, suggesting a potential competitive inhibition by these molecules. Additionally, it is noteworthy that the structure of pitholide B (**36**), which exhibits the best binding energy and the highest number of contacts with the site (Figure 2B), is very similar to the co‐crystallized autoinducer 3‐oxo‐C_12_‐HSL in the model used for this enzyme [24].

Similarly, fuscin (**13**), neosartorin (**18**), and penicillic acid (**26**), along with the positive control, revealed interactions in the ligand‐binding site in the LBD of RhlR. In this case, neosartorin (**18**) showed the best binding energy and the highest number of contacts with the protein (Figure 2C). However, this could be due to its larger molecular size compared to the other three compounds.

Table 3 displays the interaction fractions of hydrogen bonds (IF_HB_) and hydrophobic interactions (IF_HI_) for each ligand selected during the last 50 ns of the simulation. For LasR, the compounds that showed the lowest IF_HB_ were penicillin acid (**26**), followed by 8ʹ‐hydroxyzearalenone (**21**) with residues R61 and Y56, respectively. Interestingly, (**21**) showed a similar IF_HB_ compared to the positive control with residue Y47. In contrast, the highest IF_HB_ was observed for neosartorin (**18**), 5,6‐dehydroxypenicillic acid (**27**), and citrinin (**38**). The highest IF_HI_ with LasR W88 was for neosartorin (**18**), 8ʹ‐hydroxyzearalenone (**21**), 5,6‐dehydroxypenicillic acid (**27**), pitholide B (**36**), and citrinin (**38**). It is important to note that in all cases, it was observed that the compounds could interact with residues in the binding site of LasR [37]. These results indicate that the inhibition of this QS molecular target could be directly related to the inhibition of the virulence factors (exoproteases and biofilm production) [9, 12] that are regulated by this protein.

For RhlR, the highest IF_HB_ occurred with 8ʹ‐hydroxyzearalenone (**21**) with residues D81 and W68, penicillic acid (**26**) with residues W68 and S135, and 5,6‐dehydroxypenicillic acid (**27**) with S135. On the other hand, the lowest IF_HB_ was observed for pitholide B with residue Q73. Respect to IF_HI_ with RhlR, fuscin (**13**), neosartorin (**18**), and 8ʹ‐hydroxyzearalenone (**21**) showed the highest IF_HI_ with residues Y72 and V60. As with LasR, these compounds are likely to bind to the ligand‐binding site of RhlR [36], indicating inhibition of the activity of this protein and thus inhibition of pyocyanin, a virulence factor controlled by this molecular target [11].

Overall, our findings encourage further studies to explore the effects of promising compounds, such as pitholide B (**36**) and citrinin (**38**), on gene expression to confirm QS‐controlled gene inhibition and identify other potential targets. Moreover, further characterization of the antibiofilm effects of the most promising compounds, such as determining if they promote structural biofilm alterations via confocal microscopy or other suitable techniques, is also encouraged. Additionally, their toxicity and efficacy in animal models, either alone or in combination with antibiotics, should be investigated to address *P. aeruginosa* infections which represent a significant burden to global health, in that respect, possible future applications of the compounds in the clinic could include their topical use to treat skin or burn infections, their administration via inhalation to treat respiratory infections or their use as coating for catheters, prothesis or other medical devices, to prevent biofilm formation.

## 4 Conclusions

This work revealed the anti‐QS potential of several microbial metabolites, including fuscin (**13**), neosartorin (**18**), 8ʹ‐hydroxyzearalenone (**21**), penicillic acid (**26**), 5,6‐dehydroxypenicillic acid (**27**), pitholide B (**36**) and citrinin (**38**), which were able to inhibit virulence factors (pyocyanin production, exoproteases activity, and biofilm formation) of *P. aeruginosa* PA14. These compounds showed the ability to in silico bind to the ligand‐binding domain of LasR and RhlR, molecular targets that regulate QS of this bacterium, and the production of the virulence factors mentioned above, indicating that this may be the putative mechanism of action.

## Author Contributions

**Oswaldo Pablo Martínez‐Rodríguez**: writing–original draft, writing‐review and editing, methodology, investigation, data curation, formal analysis, writing–original draft, writing–review and editing, methodology, investigation, data curation, and formal analysis. **Rodolfo García‐Contreras**: writing, review, and editing, investigation, resources, and formal analysis. **Rodrigo Aguayo‐Ortiz**: writing–review and editing, investigation, resources, software, and formal analysis. **Itzel Rubí Yeverino**: writing–review and editing, methodology, investigation, data curation, and formal analysis. **Carlos A. Fajardo‐Hernández**: writing–review and editing, methodology, investigation, data curation, and formal analysis. **Albert D. Patiño**: writing–review and editing, methodology, investigation, data curation, and formal analysis. **Hugo A. Hernández Pérez**: writing–review and editing, methodology, investigation, data curation, and formal analysis. **Mariel Hernández‐Garnica**: writing–original draft, writing–review and editing, methodology, investigation, data curation, and formal analysis. **Mario Figueroa**: writing (original draft, review, and editing), visualization, validation, resources, project administration, funding acquisition, formal analysis, and conceptualization.

## Conflicts of Interest

The authors declare no conflicts of interest.

## Supporting information




**Supporting File 1**: cbdv70555‐sup‐0001‐SuppMat.pdf

## Data Availability

The data that support the findings of this study are available in the Supporting Information of this article.
