# Supplementary material for: Natural Products Inhibit Bacterial Growth and Modulate Quorum‐Sensing Systems Las and Rhl in Pseudomonas aeruginosa PA14
Source: Chem Biodivers. 2025 Oct 3;22(12):e02040. doi: 10.1002/cbdv.202502040 (PMC12715988; doi:10.1002/cbdv.202502040)
Supplement: Supplementary file 1 — Supporting File 1: cbdv70555‐sup‐0001‐SuppMat.pdf [file CBDV-22-e02040-s001.pdf]

# Supporting Information

**Supplementary Table S1.** NPs library evaluated against growth and virulence factors controlled by *P. aeruginosa* PA14 quorum sensing.

| | | | |
|---|---|---|---|
| 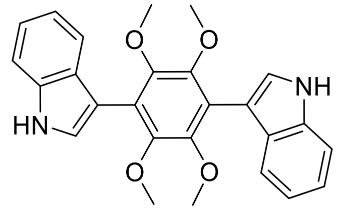 Asterriquinol D dimethyl ether (**1**) | 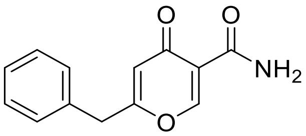 Carbonarone A (**2**) | 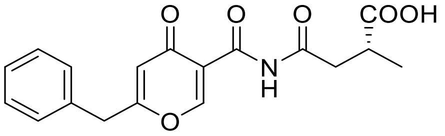 Epi-pestalamide A (**3**) | 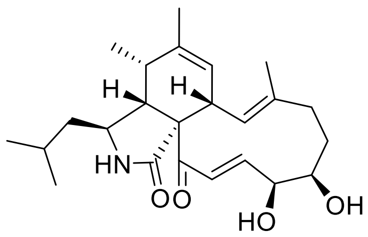 Aspochalasin D (**4**) |
| 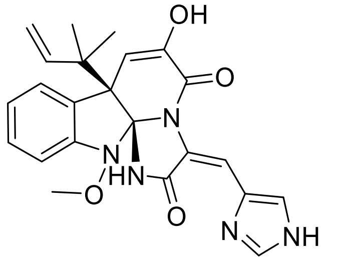 Meleagrin A (**5**) | 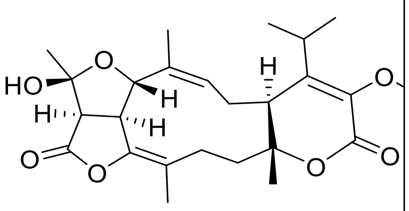 Atranone B (**6**) | 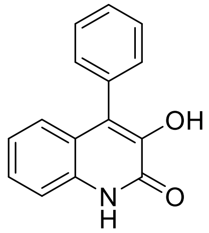 Viridicatin (**7**) | 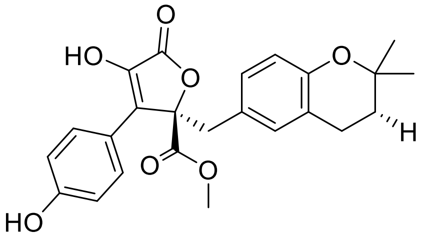 Aspernolide A (**8**) |
| 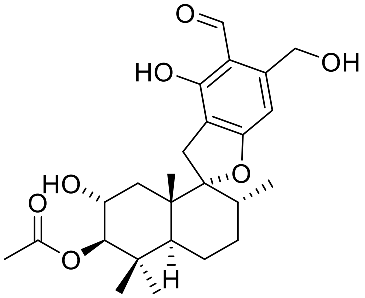 Myrotecisin B (**9**) | 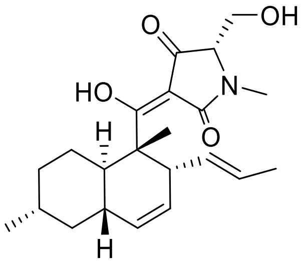 Equisetin (**10**) | 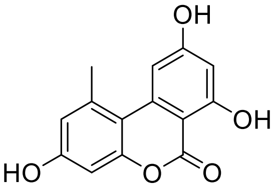 Alternariol (**11**) | 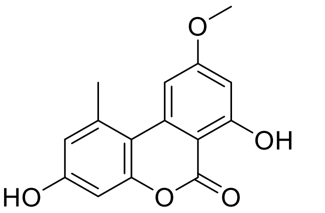 Alternariol 4-methyl ether (**12**) |

| | | | |
|---|---|---|---|
| 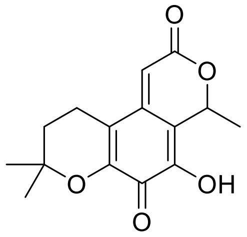 Fuscin (**13**) | 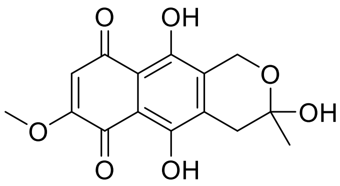 Fusarubin (**14**) | 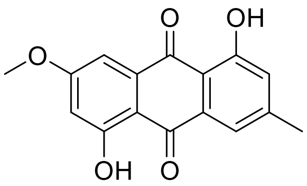 Przwalsquinone B (**15**) | 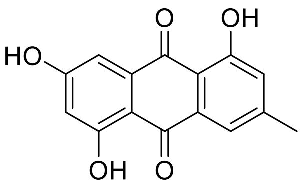 Alatonin (**16**) |
| 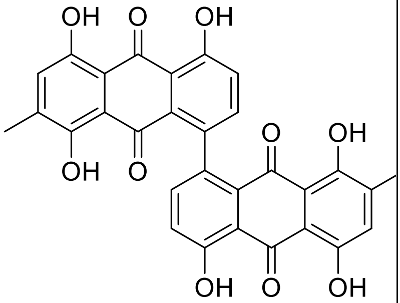 Iridoskyrin (**17**) | 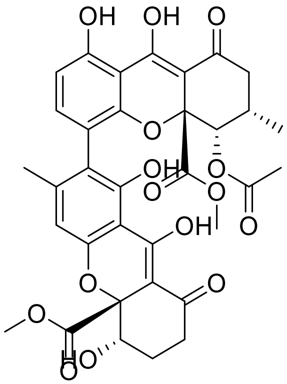 Neosartorin (**18**) | 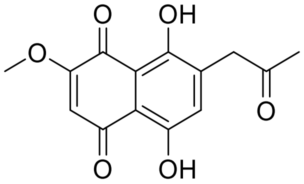 Javanicin (**19**) | 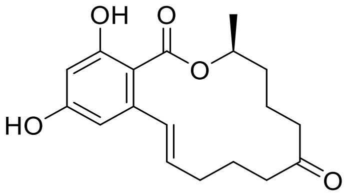 Zearalenone (**20**) |
| 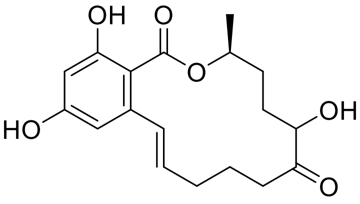 8'-Hydroxyzearalenone (**21**) | 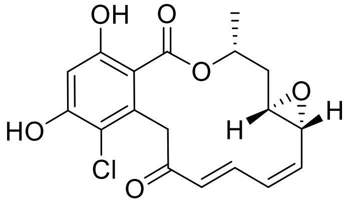 Monorden A (**22**) | 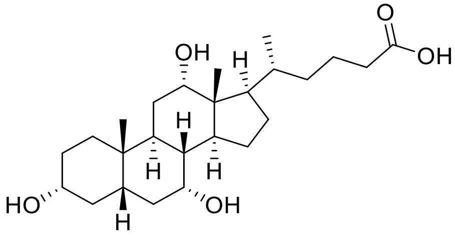 Cholic acid (**23**) | 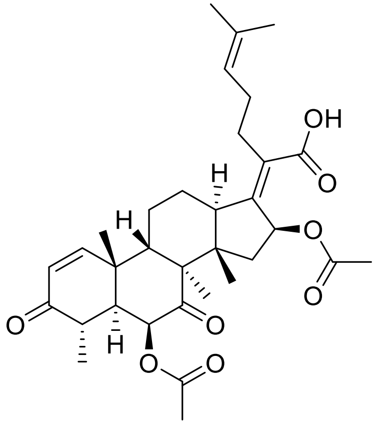 Helvolic acid (**24**) |

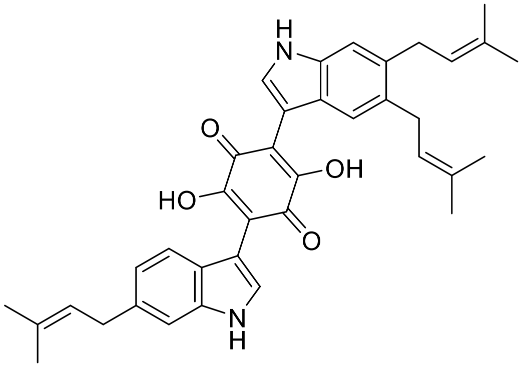


Cochliodinol (**25**)

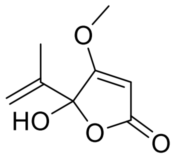


Penicillic acid (**26**)

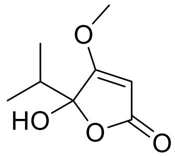


5,6-Dihydropenicillic acid (**27**)

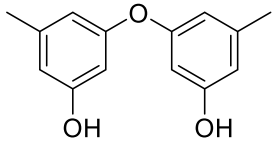


Diorcinol (**28**)

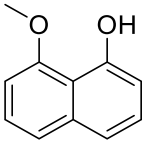


8-Methoxynaphthalene-1-ol (**29**)

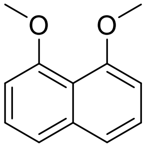


1,8-Dimethoxynaphthalen (**30**)

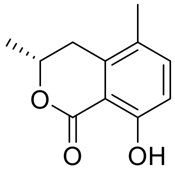


5-Methylmellein (**31**)

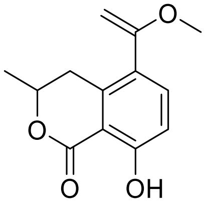


5-Carboxy-methylmellein (**32**)

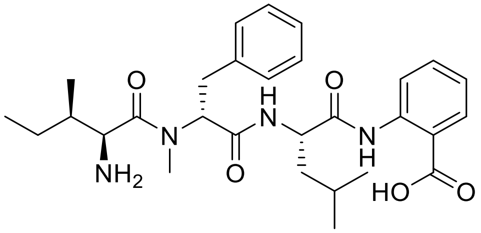


Hirsutellic acid (**33**)

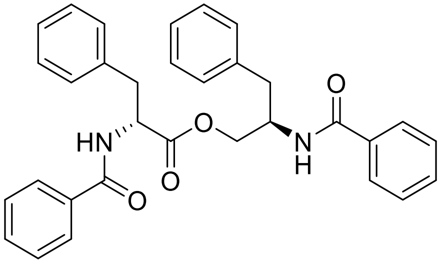


Asperphenamate (**34**)

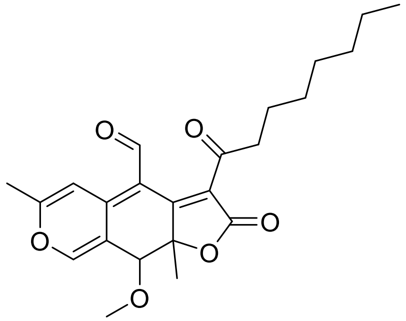


9-Methoxy-6,9a-dimethyl-3-octanoyl-2-oxo-9,9a-dihydro-2*H*-furo[3,2-*g*]benzopiran-4-carbaldehyde (**35**)

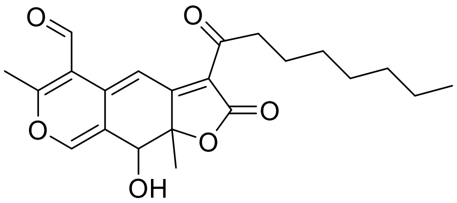

Pitholide B (**36**)

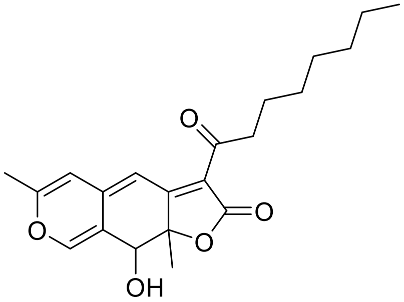

Pitholide D (**37**)

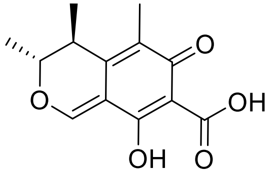

Citrinin (**38**)

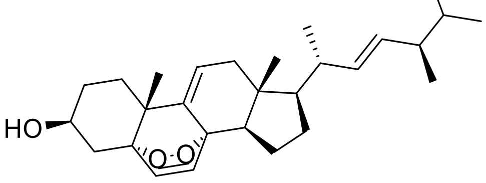

5,8-Epi-Dioxyergosta-6,9(11),22-trien-3-ol (**39**)

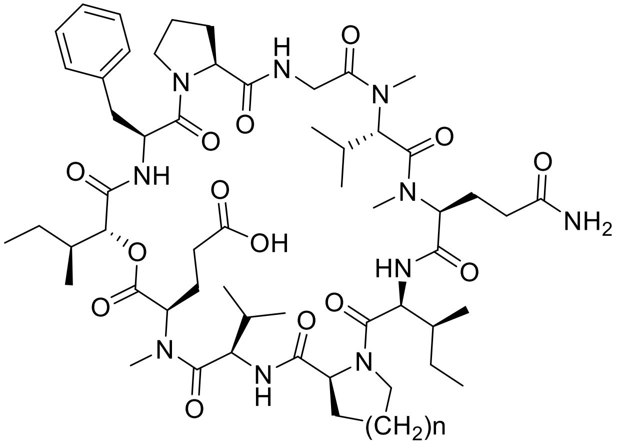

Sch-378161, n=1 (**40**)
Sch-217048, n=2 (**41**)

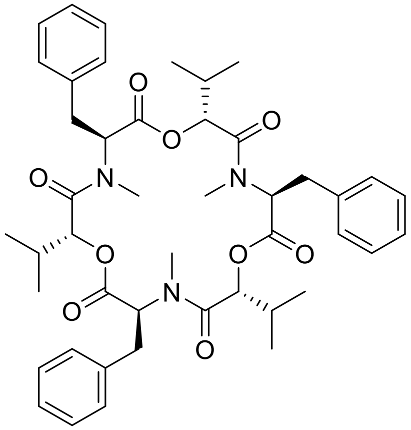

Beauvericin (**42**)

**Supplementary Table S2.** Results of antimicrobial activity and virulence factor inhibition of the NPs library against *P. aeruginosa* PA14.

| Compound | % Inhibition ± SD | | | | | | | |
|---|---|---|---|---|---|---|---|---|
| | Growth | | Pyocyanin | | Exoproteases | | Biofilms | |
| | 16 µg/mL | 64 µg/mL | 16 µg/mL | 64 µg/mL | 16 µg/mL | 64 µg/mL | 16 µg/mL | 64 µg/mL |
| LB (negative control) | 0.00±3.26 | | 0.00±1.85 | | 0.0±3.23 | | 0.00±2.70 | |
| DMSO 1.6% (vehicle) | 1.21±5.20 | | 1.39±3.17 | | 0.83±1.41 | | 0.49±1.48 | |
| Asterriquinol D dimethyl ether (**1**) | 1.08±3-64 | 37.50±3.75 | 41.05±5.10 | 59.88±1.07 | 23.90±3.60 | 34.22±2.39 | 34.16±3.14 | 53.79±3.54 |
| Carbonarone A (**2**) | -6.72±9.85 | 44.09±4.44 | 32.10±5.11 | 61.11±4.63 | 14.06±7.04 | 40.75±3.55 | 47.32±2.88 | 46.12±3.25 |
| Epi-pestalamide A (**3**) | 34.21±2.22 | 40.05±4.03 | 44.14±5.10 | 65.12±5.43 | 62.28±2.23 | 48.04±4.02 | 58.12±5.76 | 48.49±13.29 |
| Aspochalasin D (**4**) | 32.53±0.62 | 39.11±2.36 | 54.32±5.10 | 65.43±2.33 | 52.03±4.20 | 63.22±0.86 | 30.87±7.33 | 70.42±4.13 |
| Meleagrin A (**5**) | 30.09±1.87 | 43.89±5.11 | 19.27±3.25 | 67.71±5.02 | -0.59±3.02 | 63.22±3.71 | 7.67±1.77 | 47.98±2.46 |
| Atranone B (**6**) | 34.90±2.67 | 57.07±1.75 | 50.00±1.56 | 80.73±5.50 | 7.41±0.62 | 71.11±0.53 | 16.42±5.44 | 60.67±4.07 |
| Viridicatin (**7**) | 13.64±6.04 | 30.52±0.88 | 50.48±4.35 | 71.06±5.79 | 9.12±3.89 | 50.05±7.38 | 13.64±6.04 | 18.55±3.24 |
| Aspernolide A (**8**) | 15.63±3.08 | 28.60±2.78 | 8.56±4.38 | 29.79±4.63 | 21.33±2.78 | 48.04±1.45 | 15.41±4.89 | 46.23±4.86 |
| Myrotecisin B (**9**) | 17.70±0.64 | 34.97±1.06 | 14.72±4.57 | 45.18±3.05 | 69.66±3.02 | 71.11±1.87 | 14.46±2.41 | 19.68±3.03 |
| Equisetin (**10**) | -20.37±6.74 | 28.51±9.27 | 19.80±3.52 | 61.42±2.33 | 60.34±6.29 | 34.22±9.04 | 58.50±2.45 | 59.84±3.29 |
| Alternariol (**11**) | -68.82±6.12 | 13.48±1.06 | 11.68±2.64 | 65.48±0.88 | 44.86±1.84 | 40.75±7.49 | 10.84±4.42 | 30.92±4.23 |
| Alternariol 4-methyl ether (**12**) | 52.35±0.76 | 64.96±0.76 | 33.72±4.36 | 68.09±1.47 | 6.24±0.62 | 26.38±0.62 | -18.85±3.14 | -34.06±3.54 |
| Fuscin (**13**) | 8.43±2.74 | 53.09±8.22 | 19.29±1.52 | 66.50±3.05 | 65.81±0.67 | 48.04±5.30 | 50.74±1.81 | 58.77±2.21 |
| Fusarubin (**14**) | -32.02±6.95 | 32.02±0.64 | 1.02±5.49 | 73.10±2.33 | 45.92±1.59 | 50.05±4.12 | 59.97±3.80 | 70.95±4.26 |
| Przwalsquinone B (**15**) | 17.37±8.35 | 39.75±2.22 | 32.32±6.14 | 21.89±5.56 | 37.97±1.85 | 71.11±2.72 | 59.26±11.26 | 31.86±8.10 |
| Alatonin (**16**) | 18.53±2.22 | 24.46±1.92 | 20.58±3.99 | 61.32±3.77 | 10.54±2.21 | 63.22±1.35 | 20.58±3.97 | 61.32±3.77 |
| Iridoskyrin (**17**) | 43.95±2.90 | 54.43±3.08 | 50.68±3.08 | 59.93±4.11 | 50.27±2.81 | 34.22±2.13 | 50.68±3.08 | 64.38±3.61 |
| Neosartorin (**18**) | 8.50±3.12 | 48.31±5.29 | 40.74±3.70 | 45.68±4.44 | 24.34±6.61 | 71.11±3.37 | 40.74±3.70 | 45.68±4,45 |
| Javanicin (**19**) | 6.96±1.71 | 35.82±4.33 | 18.93±1.89 | 39.51±3.80 | 22.63±2.74 | 40.75±4.55 | 18.93±1.89 | 39.51±3.70 |
| Zearalenone (**20**) | 8.29±1.75 | 24.05±2.00 | 6.58±4.99 | 44.03±3.56 | 5.35±2.67 | 48.04±4.25 | 6.58±4.99 | 44.03±3.56 |
| 8'-Hydroxyzearalenone (**21**) | 5.02±1.80 | 41.97±1.34 | 32.10±3.27 | 55.56±1.23 | 2.89±1.74 | 50.05±4.43 | 32.10±3.27 | 55.56±1.23 |
| Monorden A (**22**) | 20.74±4.54 | 40.69±3.34 | 27.08±1.78 | 70.31±1.56 | 0.53±0.63 | 34.22±5.00 | 34.51±3.24 | 52.31±2.51 |
| Cholic acid (**23**) | 25.02±2.40 | 54.40±2.02 | 37.50±3.13 | 75.52±2.39 | 3.71±2.06 | 40.75±0.10 | 63.82±1.33 | 60.87±2.47 |
| Helvolic acid (**24**) | 21.83±3.03 | 22.40±3.44 | 35.96±5.27 | 47.95±6.84 | 8.11±1.66 | 50.05±1.66 | 35.96±5.27 | 59.93±1.78 |
| Cochliodinol (**25**) | 18.85±4.95 | 30.45±0.14 | 26.26±2.67 | 39.06±8.16 | 16.27±5.34 | 34.22±1.31 | 48.15±4.90 | 56.86±11.89 |
| Penicillic acid (**26** | 2.26±0.88 | 33.46±1.02 | 37.30±2.89 | 68.17±0.96 | 16.23±5.40 | 63.22±3.58 | 31.70±0.88 | 48.28±1.19 |
| 5,6-Dihydropenicillic acid (**27**) | 3.73±5.00 | 9.91±2.06 | 21.86±0.96 | 52.41±5.89 | 8.05±6.10 | 71.11±2.50 | 26.62±12.10 | 56.13±1.84 |
| Diorcinol (**28**) | 21.56±4.22 | 20.58±2.84 | 16.50±2.54 | 11.78±6.57 | 5.80±0.57 | 48.04±7.40 | 30.25±4.66 | 17.16±9.57 |

| | | | | | | | | |
|---|---|---|---|---|---|---|---|---|
| 8-Methoxynaphthalene-1-ol (**29**) | 34.49±6.38 | 43.30±1.78 | 15.23±1.89 | 59.26±1.23 | 28.95±3.68 | 40.75±3.43 | 15.23±1.89 | 59.26±1.23 |
| 1,8-Dimethoxynaphthalen (**30**) | 21.72±2.78 | 48.07±2.00 | 31.25±3.15 | 79.69±1.56 | 0.29±3.72 | 48.04±1.59 | 45.72±4.13 | 78.47±3.98 |
| 5-Methylmellein (**31**) | 15.51±1.77 | 17.37±1.33 | 9.32±4.20 | 46.62±1.47 | 11.38±4.68 | 34.22±4.68 | 15.51±1.77 | 40.73±2.50 |
| 5-Carboxy-methylmellein (**32**) | 38.49±1.51 | 51.09±1.51 | 19.48±3.44 | 53.85±1.29 | 40.87±1.99 | 45.57±3.37 | 55.92±9.85 | 73.51±12.02 |
| Hirsutellic acid (**33**) | 25.68±2.58 | 36.63±0.51 | 42.09±4.77 | 40.74±4.55 | 13.90±3.56 | 40.75±1.27 | 77.16±8.75 | 59.31±6.63 |
| Asperphenamate (**34**) | -11.16±2.02 | 28.90±3.83 | 33.33±5.16 | 63.58±5.66 | 31.10±4.93 | 50.05±0.99 | 34.34±2.36 | 54.79±2.66 |
| 9-Methoxy-6,9a-dimethyl-3-octanoyl-2-oxo-9,9a-dihydro-2*H*-furo[3,2-*g*]benzopiran-4-carbaldehyde (**35**) | 36.89±5.17 | 49.50±5.17 | 25.70±2.21 | 74.80±2.21 | 14.97±3.83 | 33.76±4.36 | 42.46±7.29 | 63.51±10.92 |
| Pitholide B (**36**) | 5.83±4.19 | 24.97±7.37 | 33.74±4.67 | 70.37±4.54 | 18.03±0.56 | 34.22±3.39 | 33.74±4.67 | 70.37±4.45 |
| Pitholide D(**37**) | 30.41±3.72 | 47.09±2.57 | 33.90±3.30 | 51.03±4.63 | 17.41±1.23 | 71.11±1.23 | 23.63±3.30 | 44.18±3.30 |
| Citrinin (**38**) | 12.11±1.68 | 26.50±2.99 | 60.62±4.63 | 63.36±2.14 | 33.90±1.77 | 63.22±2.13 | 64.04±2.72 | 74.32±2.72 |
| 5,8-Epi-dioxyergosta-6,9(11),22-trien-3-ol (**39**) | 70.50±9.72 | 83.46±5.17 | 53.85±2.14 | 53.85±2.14 | 17.92±1.21 | 24.63±2.26 | 82.46±7.29 | 89.51±10.92 |
| Sch-378161 (**40**) | 29.71±1.45 | 35.64±2.59 | 26.26±2.02 | 11.45±8.23 | 18.33±3.67 | 50.05±2.25 | 48.15±3.70 | 16.67±11.98 |
| Sch-217048 (**41**) | 17.12±4.01 | 28.89±2.78 | 41.75±7.09 | 46.13±7.45 | 16.65±1.13 | 63.22±7.13 | 76.54±13.01 | 67.16±10.84 |
| Beauvericin (**42**) | 28.40±4.29 | 40.17±4.53 | 7.36±3.69 | 60.23±0.49 | 19.73±3.95 | 29.80±3.95 | 45.31±9.33 | 59.20±5.78 |
| Quercetin 50 μM (positive control) | 2.96±2.47 | | 73.40±3.82 | | 80.30±0.76 | | 85.29±8.24 | |

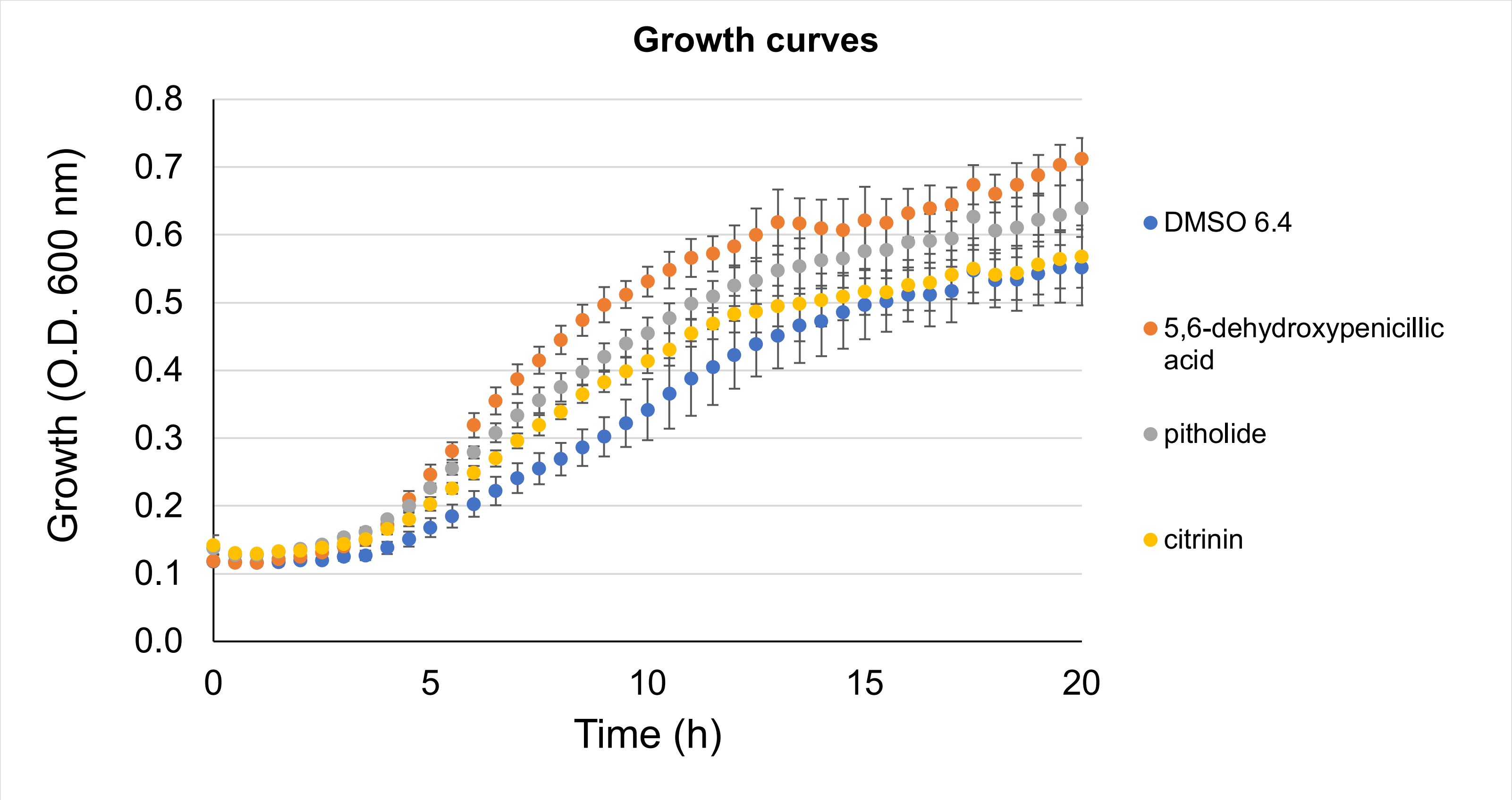


**Supplementary Figure S1.** Growth curves of PA14 in the presence of DMSO (compound vehicle) and 5,6-dehydroxypenicillic acid (**27**), pitholide B (**36**), and citrinin (**38**), at a concentration of 64 μg/mL. Data represents the mean of three independent PA14 cultures per compound ± standard deviation.

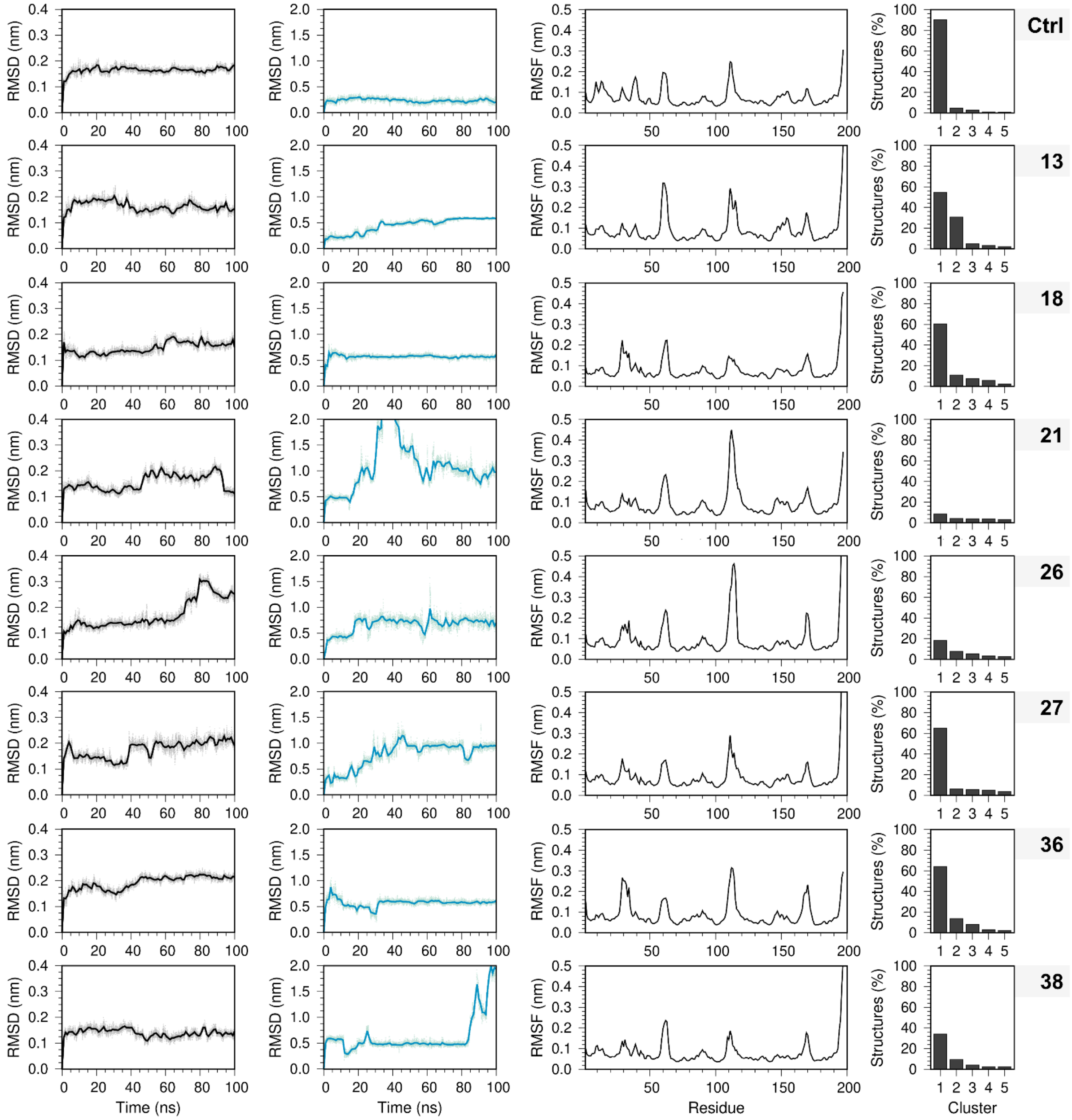


**Supplementary Figure S2.** Analysis of MD simulations for the PaLasI-ligand complexes. The figure shows the root-mean-square deviation (RMSD) of both the protein backbone (in black) and the ligand (in blue) over the course of the 100 ns MD simulations for each ligand. Additionally, it covers the per-residue root-mean-square fluctuation (RMSF) and the percentage of structures within the five most densely populated clusters from the final 50 ns of the MD simulations.

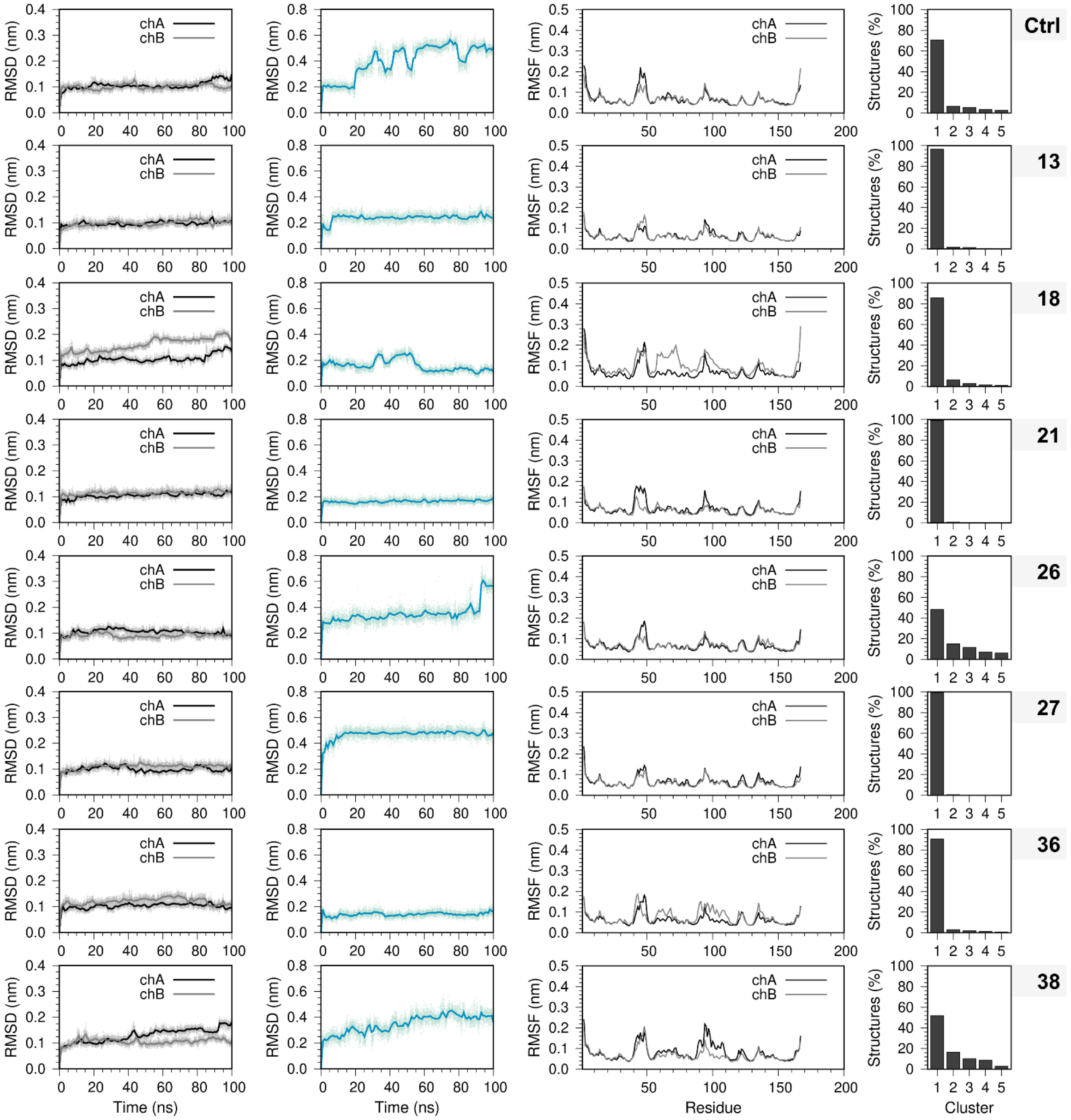


**Supplementary Figure S3.** Analysis of MD simulations for the PaLasI-ligand complexes. The figure shows the root-mean-square deviation (RMSD) of both the protein backbone (in black) and the ligand (in blue) over the course of the 100 ns MD simulations for each ligand. Additionally, it covers the per-residue root-mean-square fluctuation (RMSF) and the percentage of structures within the five most densely populated clusters from the final 50 ns of the MD simulations.

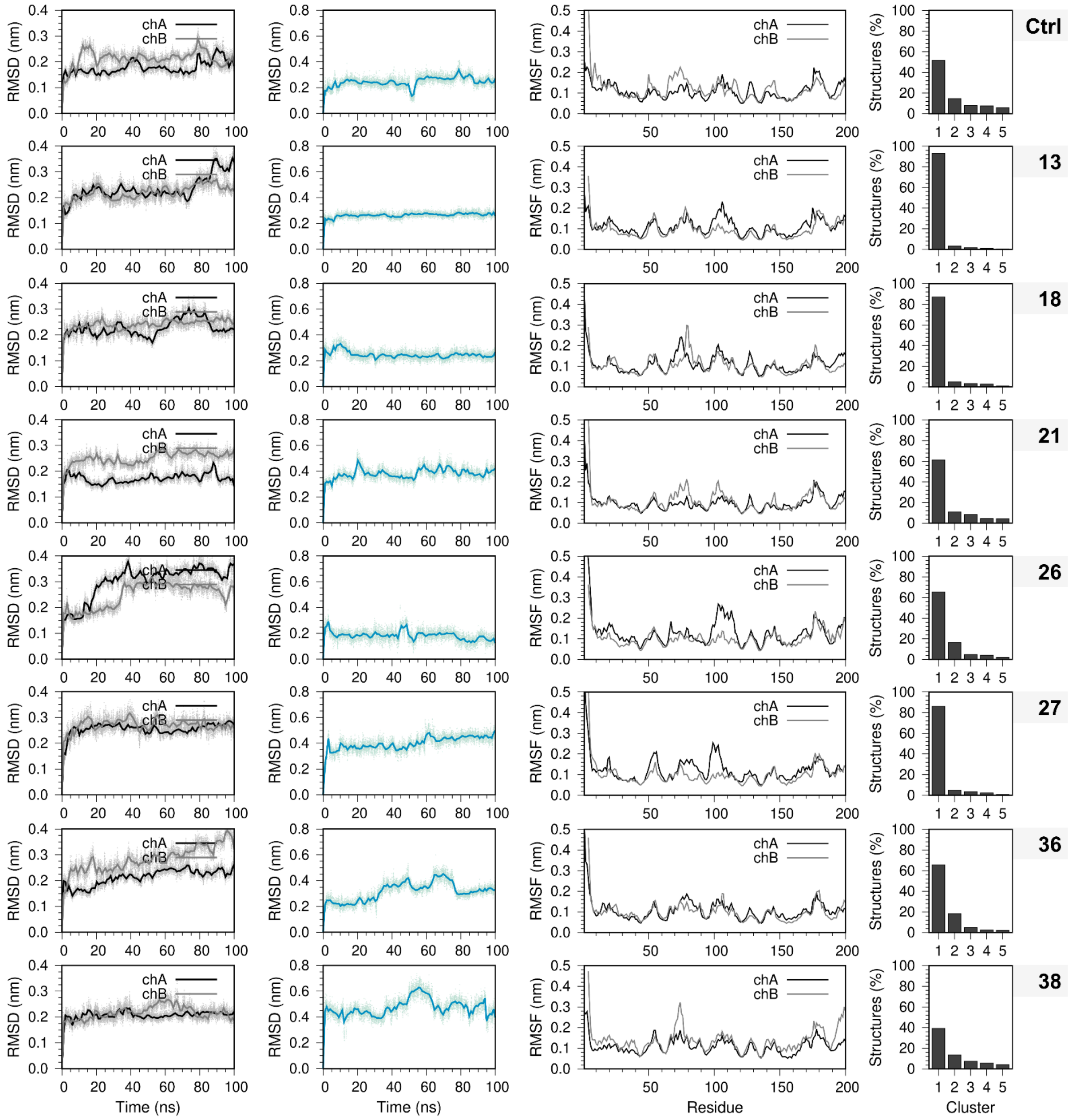


**Supplementary Figure S4.** Analysis of MD simulations for the PaRhlR-ligand complexes. The figure shows the root-mean-square deviation (RMSD) of both the protein backbone (in black) and the ligand (in blue) over the course of the 100 ns MD simulations for each ligand. Additionally, it covers the per-residue root-mean-square fluctuation (RMSF) and the percentage of structures within the five most densely populated clusters from the final 50 ns of the MD simulations.
